# Classical Human Leukocyte Antigen Alleles and C4 Haplotypes Are Not Significantly Associated With Depression

**DOI:** 10.1016/j.biopsych.2019.06.031

**Published:** 2020-03-01

**Authors:** Kylie P. Glanville, Jonathan R.I. Coleman, Ken B. Hanscombe, Jack Euesden, Shing Wan Choi, Kirstin L. Purves, Gerome Breen, Tracy M. Air, Till F.M. Andlauer, Bernhard T. Baune, Elisabeth B. Binder, Douglas H.R. Blackwood, Dorret I. Boomsma, Henriette N. Buttenschøn, Lucía Colodro-Conde, Udo Dannlowski, Nese Direk, Erin C. Dunn, Andreas J. Forstner, Eco J.C. de Geus, Hans J. Grabe, Steven P. Hamilton, Ian Jones, Lisa A. Jones, James A. Knowles, Zoltán Kutalik, Douglas F. Levinson, Glyn Lewis, Penelope A. Lind, Susanne Lucae, Patrik K. Magnusson, Peter McGuffin, Andrew M. McIntosh, Yuri Milaneschi, Ole Mors, Sara Mostafavi, Bertram Müller-Myhsok, Nancy L. Pedersen, Brenda W.J.H. Penninx, James B. Potash, Martin Preisig, Stephan Ripke, Jianxin Shi, Stanley I. Shyn, Jordan W. Smoller, Fabian Streit, Patrick F. Sullivan, Henning Tiemeier, Rudolf Uher, Sandra Van der Auwera, Myrna M. Weissman, Major Depressive Disorder Working Group of the Psychiatric Genomics Consortium, Naomi R. Wray, Stephan Ripke, Manuel Mattheisen, Maciej Trzaskowski, Enda M. Byrne, Abdel Abdellaoui, Mark J. Adams, Esben Agerbo, Tracy M. Air, Till F.M. Andlauer, Silviu-Alin Bacanu, Marie Bækvad-Hansen, Aartjan T.F. Beekman, Tim B. Bigdeli, Elisabeth B. Binder, Julien Bryois, Henriette N. Buttenschøn, Jonas Bybjerg-Grauholm, Na Cai, Enrique Castelao, Jane Hvarregaard Christensen, Toni-Kim Clarke, Jonathan R.I. Coleman, Lucía Colodro-Conde, Baptiste Couvy-Duchesne, Nick Craddock, Gregory E. Crawford, Gail Davies, Ian J. Deary, Franziska Degenhardt, Eske M. Derks, Nese Direk, Conor V. Dolan, Erin C. Dunn, Thalia C. Eley, Valentina Escott-Price, Farnush Farhadi Hassan Kiadeh, Hilary K. Finucane, Jerome C. Foo, Andreas J. Forstner, Josef Frank, Héléna A. Gaspar, Michael Gill, Fernando S. Goes, Scott D. Gordon, Jakob Grove, Lynsey S. Hall, Christine Søholm Hansen, Thomas F. Hansen, Stefan Herms, Ian B. Hickie, Per Hoffmann, Georg Homuth, Carsten Horn, Jouke-Jan Hottenga, David M. Hougaard, David M. Howard, Marcus Ising, Rick Jansen, Ian Jones, Lisa A. Jones, Eric Jorgenson, James A. Knowles, Isaac S. Kohane, Julia Kraft, Warren W. Kretzschmar, Zoltán Kutalik, Yihan Li, Penelope A. Lind, Donald J. MacIntyre, Dean F. MacKinnon, Robert M. Maier, Wolfgang Maier, Jonathan Marchini, Hamdi Mbarek, Patrick McGrath, Peter McGuffin, Sarah E. Medland, Divya Mehta, Christel M. Middeldorp, Evelin Mihailov, Yuri Milaneschi, Lili Milani, Francis M. Mondimore, Grant W. Montgomery, Sara Mostafavi, Niamh Mullins, Matthias Nauck, Bernard Ng, Michel G. Nivard, Dale R. Nyholt, Paul F. O'Reilly, Hogni Oskarsson, Michael J. Owen, Jodie N. Painter, Carsten Bøcker Pedersen, Marianne Giørtz Pedersen, Roseann E. Peterson, Erik Pettersson, Wouter J. Peyrot, Giorgio Pistis, Danielle Posthuma, Jorge A. Quiroz, Per Qvist, John P. Rice, Brien P. Riley, Margarita Rivera, Saira Saeed Mirza, Robert Schoevers, Eva C. Schulte, Ling Shen, Jianxin Shi, Stanley I. Shyn, Engilbert Sigurdsson, Grant C.B. Sinnamon, Johannes H. Smit, Daniel J. Smith, Hreinn Stefansson, Stacy Steinberg, Fabian Streit, Jana Strohmaier, Katherine E. Tansey, Henning Teismann, Alexander Teumer, Wesley Thompson, Pippa A. Thomson, Thorgeir E. Thorgeirsson, Matthew Traylor, Jens Treutlein, Vassily Trubetskoy, Andrés G. Uitterlinden, Daniel Umbricht, Sandra Van der Auwera, Albert M. van Hemert, Alexander Viktorin, Peter M. Visscher, Yunpeng Wang, Bradley T. Webb, Shantel Marie Weinsheimer, Jürgen Wellmann, Gonneke Willemsen, Stephanie H. Witt, Yang Wu, Hualin S. Xi, Jian Yang, Futao Zhang, Volker Arolt, Bernhard T. Baune, Klaus Berger, Dorret I. Boomsma, Sven Cichon, Udo Dannlowski, EJC. de Geus, J. Raymond DePaulo, Enrico Domenici, Katharina Domschke, Tõnu Esko, Hans J. Grabe, Steven P. Hamilton, Caroline Hayward, Andrew C. Heath, Kenneth S. Kendler, Stefan Kloiber, Glyn Lewis, Qingqin S. Li, Susanne Lucae, Pamela AF. Madden, Patrik K. Magnusson, Nicholas G. Martin, Andrew M. McIntosh, Andres Metspalu, Ole Mors, Preben Bo Mortensen, Bertram Müller-Myhsok, Merete Nordentoft, Markus M. Nöthen, Michael C. O'Donovan, Sara A. Paciga, Nancy L. Pedersen, Brenda W.J.H. Penninx, Roy H. Perlis, David J. Porteous, James B. Potash, Martin Preisig, Marcella Rietschel, Catherine Schaefer, Thomas G. Schulze, Jordan W. Smoller, Kari Stefansson, Henning Tiemeier, Rudolf Uher, Henry Völzke, Myrna M. Weissman, Thomas Werge, Cathryn M. Lewis, Douglas F. Levinson, Gerome Breen, Anders D. Børglum, Patrick F. Sullivan, Paul F. O'Reilly, Cathryn M. Lewis

**Affiliations:** 1 Social Genetic and Developmental Psychiatry Centre, Institute of Psychiatry, Psychology and Neuroscience, King's College London, London, United Kingdom; 2 National Institute for Health Research Biomedical Research Centre South London and Maudsley National Health Service Trust, King's College London, London, United Kingdom; 3 Department of Medical and Molecular Genetics, King's College London, London, United Kingdom; 4 Division of Psychiatry, University College London, London, United Kingdom; 5 Division of Psychiatry, University of Edinburgh, Edinburgh, United Kingdom; 6 Centre for Cognitive Ageing and Cognitive Epidemiology, University of Edinburgh, Edinburgh, United Kingdom; 7 Medical Research Council Centre for Neuropsychiatric Genetics and Genomics, Cardiff University, Cardiff, United Kingdom; 8 Department of Psychological Medicine, University of Worcester, Worcester, United Kingdom; 9 University of Liverpool, Liverpool, United Kingdom; 10 Genetics and Genomic Sciences, Icahn School of Medicine, Mount Sinai, New York, New York; 11 Division of Epidemiology, New York State Psychiatric Institute, New York, New York; 12 Department of Psychiatry, Columbia University College of Physicians and Surgeons, New York, New York; 13 Department of Psychiatry and Behavioral Sciences, Emory University School of Medicine, Emory University, Atlanta, Georgia; 14 Department of Psychiatry, Massachusetts General Hospital, Boston, Massachusetts; 15 Psychiatric and Neurodevelopmental Genetics Unit, Massachusetts General Hospital, Boston, Massachusetts; 16 Analytic and Translational Genetics Unit, Massachusetts General Hospital, Boston, Massachusetts; 17 Stanley Center for Psychiatric Research, Broad Institute, Cambridge, Massachusetts; 18 Department of Medical and Population Genetics, Broad Institute, Cambridge, Massachusetts; 19 Department of Psychiatry, Kaiser Permanente Northern California, San Francisco, California; 20 Psychiatry and the Behavioral Sciences, University of Southern California, Los Angeles, California; 21 Psychiatry and Behavioral Sciences, Stanford University, Stanford, California; 22 Psychiatry, University of Iowa, Iowa City, Iowa; 23 Division of Cancer Epidemiology and Genetics, National Cancer Institute, Bethesda, Maryland; 24 Behavioral Health Services, Kaiser Permanente Washington, Seattle, Washington; 25 Department of Genetics, University of North Carolina at Chapel Hill, Chapel Hill, North Carolina; 26 Department of Psychiatry, University of North Carolina at Chapel Hill, Chapel Hill, North Carolina; 27 Discipline of Psychiatry, Adelaide Medical School, University of Adelaide, Adelaide, South Australia, Australia; 28 Department of Psychiatry, Melbourne Medical School, University of Melbourne, Melbourne, Victoria, Australia; 29 Florey Institute for Neuroscience and Mental Health, University of Melbourne, Melbourne, Victoria, Australia; 30 Genetics and Computational Biology, QIMR Berghofer Medical Research Institute, Brisbane, Queensland, Australia; 31 Department of Translational Research in Psychiatry, Max Planck Institute of Psychiatry, Münster, Germany; 32 Munich Cluster for Systems Neurology (SyNergy), Münster, Germany; 33 Department of Psychiatry, University of Münster, Münster, Germany; 34 Department of Psychiatry, University of Münster, Münster, Germany; 35 Institute of Human Genetics, School of Medicine and University Hospital Bonn, University of Bonn, Bonn, Germany; 36 Centre for Human Genetics, University of Marburg, Marburg, Germany; 37 Department of Psychiatry and Psychotherapy, University Medicine Greifswald, Greifswald, Germany; 38 Max Planck Institute of Psychiatry, Munich, Germany; 39 Department of Psychiatry and Psychotherapy, Universitätsmedizin Berlin Campus Charité Mitte, Berlin, Germany; 40 Department of Genetic Epidemiology in Psychiatry, Central Institute of Mental Health, Medical Faculty Mannheim, Heidelberg University, Mannheim, Germany; 41 Department of Biological Psychology and EMGO+ Institute for Health and Care Research, Vrije Universiteit Medical Center, Vrije Universiteit Amsterdam, Amsterdam, The Netherlands; 42 Amsterdam Public Health Institute, Vrije Universiteit Medical Center, Vrije Universiteit Amsterdam, Amsterdam, The Netherlands; 43 Department of Psychiatry, Amsterdam Universiteit Medical Center, Vrije Universiteit Amsterdam, Amsterdam, The Netherlands; 44 Epidemiology, Erasmus Medical Center, Rotterdam, The Netherlands; 45 Child and Adolescent Psychiatry, Erasmus Medical Center, Rotterdam, The Netherlands; 46 Psychiatry, Erasmus Medical Center, Rotterdam, The Netherlands; 47 NIDO | Danmark, Regional Hospital West Jutland, Herning, Denmark; 48 iPSYCH, The Lundbeck Foundation Initiative for Integrative Psychiatric Research, Denmark; 49 Psychosis Research Unit, Aarhus University Hospital, Risskov, Aarhus, Denmark; 50 Department of Psychiatry, Dokuz Eylul University School Of Medicine, Izmir, Turkey; 51 Department of Psychiatry, University of Basel, Basel, Switzerland; 52 Department of Biomedicine, University of Basel, Basel, Switzerland; 53 Institute of Social and Preventive Medicine, University Hospital of Lausanne, Lausanne, Switzerland; 54 Swiss Institute of Bioinformatics, Lausanne, Switzerland; 55 Department of Psychiatry, University Hospital of Lausanne, Prilly, Switzerland; 56 Department of Medical Epidemiology and Biostatistics, Karolinska Institutet, Stockholm, Sweden; 57 Department of Medical Genetics, University of British Columbia, Vancouver, British Columbia, Canada; 58 Department of Statistics, University of British Columbia, Vancouver, British Columbia, Canada; 59 Department of Psychiatry, Dalhousie University, Halifax, Nova Scotia, Canada

**Keywords:** Autoimmune disorder, Complement, Genetic association, Human leukocyte antigen, Major depressive disorder, Major histocompatibility complex

## Abstract

**Background:**

The prevalence of depression is higher in individuals with autoimmune diseases, but the mechanisms underlying the observed comorbidities are unknown. Shared genetic etiology is a plausible explanation for the overlap, and in this study we tested whether genetic variation in the major histocompatibility complex (MHC), which is associated with risk for autoimmune diseases, is also associated with risk for depression.

**Methods:**

We fine-mapped the classical MHC (chr6: 29.6–33.1 Mb), imputing 216 human leukocyte antigen (HLA) alleles and 4 complement component 4 (C4) haplotypes in studies from the Psychiatric Genomics Consortium Major Depressive Disorder Working Group and the UK Biobank. The total sample size was 45,149 depression cases and 86,698 controls. We tested for association between depression status and imputed MHC variants, applying both a region-wide significance threshold (3.9 × 10^−6^) and a candidate threshold (1.6 × 10^−4^).

**Results:**

No HLA alleles or C4 haplotypes were associated with depression at the region-wide threshold. HLA-B*08:01 was associated with modest protection for depression at the candidate threshold for testing in HLA genes in the meta-analysis (odds ratio = 0.98, 95% confidence interval = 0.97–0.99).

**Conclusions:**

We found no evidence that an increased risk for depression was conferred by HLA alleles, which play a major role in the genetic susceptibility to autoimmune diseases, or C4 haplotypes, which are strongly associated with schizophrenia. These results suggest that any HLA or C4 variants associated with depression either are rare or have very modest effect sizes.

Depression is a debilitating psychiatric disorder with an estimated lifetime prevalence of 15% (1), making it the leading cause of global disability (2). The disorder is characterized by heterogeneous symptom profiles (3) and variable treatment outcomes (4). Developing effective pharmaceutical treatments relies on uncovering the etiology of a disorder (5), and psychiatric genetics has made great progress toward this objective in the past decade 6, 7. Despite this progress, the underlying biology of depression is still not fully understood. Comorbid psychiatric and physical traits may indicate shared biological pathways and provide a path to uncovering the etiology of idiopathic psychiatric disorders (8). Here, we focus on comorbid autoimmune diseases and depression, consider the mechanisms that could drive the overlap, and test for evidence of shared genetic influences in the major histocompatibility complex (MHC).

Epidemiological studies indicate that individuals with a history of autoimmune disease are at greater risk of developing mood disorders compared with individuals without a history of autoimmune disease 9, 10, 11, 12. For example, a Danish Registry study (9) showed that the risk of developing a mood disorder increased following onset of any autoimmune disease (incident rate ratio = 1.45; 95% confidence interval [CI] = 1.39–1.52).

One interpretation is that the distress arising from autoimmune disorders is causal to the onset of a mood disorder. However, other evidence indicates that the relationship is bidirectional 13, 14. For example, another Danish Registry study (13) showed that individuals with depression were at increased risk for developing any autoimmune disease (incident rate ratio = 1.25, 95% CI = 1.19–1.31) and that this increase remained relatively stable across the first decade after diagnosis of depression.

There are several plausible explanations for the observed overlap between depression and autoimmunity. Shared environmental influences may increase risk for both disorders—for example, stress is a risk factor for autoimmune disease (15)—and there is a phenotypic and genetic correlation between anxiety and depression (16). Another view is that shared genetic influences act on autoimmune disease and depression through common immune pathways. Efforts to identify shared genetic influences were undertaken in a recent genome-wide association study (GWAS) of depression, using linkage disequilibrium (LD) score regression to estimate genetic correlations between depression and autoimmune diseases (17). There was no evidence for significant cross-trait correlations; the strongest correlation observed was between depression and inflammatory bowel disease (*r*_G_ = .07, *p* = .06). However, methods to detect genome-wide pleiotropy will not detect shared association at specific variants. Genetic variation in the MHC, which plays a crucial role in human immunity (18), should be thoroughly interrogated in depression.

The MHC is divided into 3 functionally distinct regions: class I and II regions contain highly polymorphic human leukocyte antigen (HLA) genes that are strongly associated with risk for autoimmune disease 19, 20, 21, and the class III region contains complement component 4 (C4) genes, which are strongly associated with risk for schizophrenia (22). Three recent GWASs indicated that genetic variation within the MHC is involved in risk for depression 17, 23, 24, with the strongest association located in the classical or extended class I region.

Highly polymorphic loci and long-range LD in the MHC complicate the interpretation of single nucleotide polymorphism (SNP) associations (17). However, imputed HLA alleles (25) and C4 haplotypes (22) can dissect SNP signal in the region with fine-mapping techniques. We used this approach to test whether genetic variation associated with autoimmune disease and schizophrenia is also associated with depression. Common SNPs in the MHC were tested to confirm that the pattern of association with depression was consistent with the pattern observed in previous GWASs and to provide a backbone of association across this region (17). We imputed HLA variants and common C4 haplotypes and tested whether these were associated with depression. We additionally extracted HLA alleles that increase risk of autoimmune diseases to test for association with depression. Finally, to explore the relationship between the association with HLA alleles and C4 haplotypes, we tested for association of depression with genetically predicted C4A brain expression and performed conditional analysis to assess evidence for association at HLA alleles and C4 haplotypes in strong LD.

To our knowledge, this is the first study to leverage imputation to interrogate the involvement of HLA alleles and C4 haplotypes in depression. Our efforts should lead to a better understanding of the role of these loci in depression and may provide insights into the mechanisms driving comorbid autoimmunity and depression.

## Methods and Materials

### Participants

Participant data came from a subset of the Major Depressive Disorder Working Group of the Psychiatric Genomics Consortium (PGC-MDD) (17) and from the UK Biobank (UKB) (26) to give a total of 131,847 individuals of European ancestry (55% female subjects, 45,149 depression cases, and 86,698 controls). Individual-level genotype and phenotype data were available for 26 PGC-MDD studies, totaling 39,145 individuals (54% female subjects, 15,805 cases, and 23,340 controls). Across the PGC-MDD studies, structured diagnostic interviews were conducted to identify case subjects with a lifetime diagnosis of MDD according to the DSM-IV (27), the ICD-9 (28), the ICD-10 (29), or the Composite International Diagnostic Interview Short Form (30). In most PGC-MDD studies, bipolar disorder, nonaffective psychosis, and substance use disorder were exclusion criteria in the cases, and controls were screened for absence of MDD and other psychiatric disorders. Ethical approvals were obtained by the principal investigators of each study, with all participants giving full informed consent.

The UKB is a prospective cohort study that has collected genotype and phenotype data for more than 500,000 individuals across the UK, between 40 and 69 years of age at recruitment (26). A total of 157,366 UKB participants completed an online mental health questionnaire, which assesses lifetime depressive disorder (31). Using the recommended mental health questionnaire scoring protocol (31), we identified 29,344 individuals with lifetime depressive disorder and 63,358 controls. Cases were excluded if they endorsed diagnosis of psychosis or bipolar disorder. Controls were excluded if they endorsed diagnosis of any psychiatric disorder in the mental health questionnaire, or self-reported depression or use of antidepressant medication at baseline and follow-up interviews, or had a mood disorder according to hospital episode statistics, or met the criteria for a mood disorder according to Smith *et al.* (32). Further details of the PGC-MDD and UKB samples are in Table S1 in Supplement 2.

### Genotyping and Quality Control

Quality control (QC) of genotype data in the 26 PGC-MDD studies was performed by the PGC Statistical Analysis Group using the ricopili pipeline (17) with the following thresholds: SNP missingness (before individual QC) < 0.05, individual missingness < 0.02, SNP missingness (after individual QC) < 0.02, deviation from heterozygosity |*F*_het_| < 0.20, Hardy-Weinberg equilibrium *p* value > 10^−10^ (cases) and *p* value > 10^−6^ (controls). After imputation with the 1000 Genomes reference panel (17), SNPs with INFO score > 0.8 and minor allele frequency (MAF) > 0.05 were retained for relatedness testing and principal component analysis. One individual from each pair with relatedness > 0.2 was removed, and only individuals of European ancestry were retained.

Using genotype data that had undergone preliminary QC by the UKB (26), we created an inclusion list of individuals of European ancestry using 4-means clustering on the first 2 principal components provided by the UKB. Using relatedness kinship (KING) estimates provided by the UKB, we removed 1 individual from each pair up to 3rd-degree relationships (KING *r*^2^ > .044) (33). In the remaining data, we applied QC with the following thresholds: SNP missingness (before individual QC) < 0.02, individual missingness < 0.02, SNP missingness (after individual QC) < 0.02, MAF > 0.01, Hardy-Weinberg equilibrium *p* value > 10^−8^. The UKB (26) imputed SNPs using the IMPUTE4 software (26) with the Haplotype Reference Consortium reference panel (34) and the UK10K Consortium reference panel (35) to produce dosage data in BGEN file format (version 1.2) (36). We extracted imputed SNPs from the classical MHC (chr6: 29,640,000–33,120,000) and converted to PLINK 2 binary format for association analyses in PLINK 2.0 (37).

### HLA Allele and C4 Haplotype Imputation

HLA alleles were imputed using genotype data from the PGC-MDD studies using the SNP2HLA software (25) with the Type 1 Diabetes Genetics Consortium reference panel (38) to produce dosage data in Beagle format (39). The Type 1 Diabetes Genetics Consortium reference panel contains MHC haplotype information to enable imputation of HLA alleles at 2-digit and 4-digit resolution in 8 HLA genes: *HLA-A*, *HLA-B,* and *HLA-C* in the classical class I MHC and *HLA-DRB1*, *HLA-DQA1*, *HLA-DQB1*, *HLA-DPA1,* and *HLA-DPB1* in the classical class II MHC.

HLA alleles were imputed in the UKB by the core analytical team using the HLA*IMP:02 software (26) with multipopulation reference panels (40). Collectively, the reference panels contained MHC haplotype information to enable imputation of HLA alleles in 11 HLA genes: *HLA-A*, *HLA-B*, and *HLA-C* in the classical class I MHC and *HLA-DRB5*, *HLA-DRB4*, *HLA-DRB3*, *HLA-DRB1*, *HLA-DQA1*, *HLA-DQB1*, *HLA-DPA1*, and *HLA-DPB1* in the classical class II MHC. Only HLA alleles at 4-digit resolution have been made available by the UKB. HLA alleles were encoded as biallelic in the PGC-MDD and UKB data such that imputed dosages referred to the presence of 0, 1, or 2 copies of each HLA allele.

C4 haplotypes were imputed using genotype data in the PGC-MDD and UKB using the SNP2HLA software (25) with the C4 reference panel developed by the McCarroll Lab (38) (http://mccarrolllab.com/wp-content/uploads/2014/12/MHC_haplotypes_CEU_HapMap3_ref_panel.bgl) to produce dosage data in Beagle format (39). The reference panel consists of SNP and C4 haplotypes within the extended MHC (25–34 Mb on chromosome 6) for 110 individuals from the HapMap CEU population. The reference panel contains 17 C4 haplotypes, defined by copy number variation of *C4A* and *C4B* genes in short and long form. Four C4 haplotypes with frequency >0.01 were retained: AL-AL, AL-BL, AL-BS, and BS (where A and B correspond to 2 isotypes of the *C4* gene and L and S correspond to the long and short forms). Of the common C4 haplotypes, 3 (AL-AL, AL-BL, and AL-BS) segregate on 2, 3, and 5 different SNP haplotypes, respectively. Association results for these C4 haplotypes were calculated by meta-analyzing across SNP haplotypes corresponding to each C4 structure.

### Statistical Analyses

In the PGC-MDD group, we tested each HLA allele and C4 haplotype for association with MDD case-control status using an additive logistic regression model applied to dosage data. We included 6 principal components to control for population structure. We extracted association results for SNPs in the classical MHC from PGC-MDD analyses in each study, applying further QC such that only variants with a MAF >0.01 and an INFO score ≥0.6 were retained. Post-QC variants were meta-analyzed across the 26 PGC-MDD studies using an inverse-variance weighted approach.

In the UKB sample, we tested each HLA allele, C4 haplotype, and imputed SNP for association with depression case-control status using an additive linear regression model applied to dosage data. We regressed 6 principal components (calculated by the UKB), batch, and center on the depression phenotype using logistic regression in R 3.4.1 (41), and used the residuals as the outcome variable in subsequent linear regression. We filtered for variants with a MAF >0.01 and an INFO score ≥0.6 before meta-analyzing across the PGC-MDD and UKB results. Analyses were performed using PLINK (version 1.9 and version 2.0) (37). Further details of QC, imputation, and analysis are given in the Supplement.

To calculate the MHC region-wide significance threshold, we used the Genetic Type I error calculator (GEC) (42), an online resource that calculates the number of effective tests by estimating LD between variants and applying a Bonferroni correction. We calculated a conservative region-wide significance threshold (3.9 × 10^−6^), controlling for all imputed SNPs in the classical MHC, and a candidate significance threshold (1.6 × 10^−4^), controlling only for HLA intragenic SNPs in the classical MHC. A summary of all analyses performed is given in Table S2 in Supplement 2.

We used the Genetic Power Calculator (43) to estimate power at the minimum and maximum INFO score thresholds. At an INFO score of 1.0, the effective sample size was 45,149 cases and 86,698 controls. For an HLA allele of frequency 0.05 (the median in our study), we had 80% power to detect an odds ratio (OR) >1.09, at a region-wide significance level of α = 3.9 × 10^−6^. At an INFO score of 0.6, the effective sample size was 27,089 cases and 52,018 controls. For an HLA allele of frequency 0.05, we had 80% power to detect an OR >1.12, at a region-wide significance level of α = 3.9 × 10^−6^.

We compared the imputation accuracy and frequency of HLA alleles and C4 haplotypes in the PGC-MDD and UKB samples for variants present in both samples. The average imputation INFO score and frequency were calculated by weighting variant INFO scores and frequencies by the effective sample size in each PGC-MDD study.

The genetic correlation between the PGC-MDD and UKB samples was calculated using the LD Score software (version 1.0.0) (44) using GWAS summary statistics for these data sets that had been previously calculated 17, 45. The local heritability of depression in the MHC was calculated using the HESS software (46), which partitions heritability into LD blocks across the genome. Using summary statistics from the PGC-MDD GWAS of depression (17) (excluding 23andMe data), we calculated the genome-wide heritability of depression and extracted the heritability estimates for the 5 LD partitions that constitute the extended MHC.

Drawing on evidence from epidemiological studies 9, 13, we identified autoimmune diseases with evidence for a bidirectional relationship with depression. We identified individuals affected by these autoimmune diseases in the UKB using hospital episode statistics and self-reported conditions. HLA risk alleles for these autoimmune diseases were identified by conducting a PubMed search using the terms “HLA” and relevant disease name. HLA alleles with evidence for independent association (*p* < 3.9 × 10^−6^) in European populations were retained. We evaluated evidence for involvement of these HLA alleles in depression, selecting those with MAF >0.05 in our study. We used the GEC (42) to determine the effective number of tests across 14 HLA alleles, and we obtained the *p* value threshold of .05/11.75 = .004.

To dissect the combined contribution of HLA alleles and C4 haplotypes to risk of depression, we performed conditional analysis of HLA alleles associated with depression and C4 haplotypes in strong LD with these variants. The LD (*r*^2^) between each common C4 haplotype (AL-AL, AL-BL, AL-BS, and BS) and all imputed HLA alleles in the UKB data set was calculated using PLINK (37).

Genetically predicted C4A brain expression was calculated for each individual. We leveraged work from Sekar *et al.* (22), who estimated the contribution of each C4 structure to C4A brain expression in postmortem brain tissue. From this model, we estimated C4A brain expression corresponding to each C4 haplotype (Table S3 in Supplement 2) and calculated individual-level C4A brain expression by multiplying the dosage for each C4 haplotype by the corresponding value for C4A brain expression. We then tested genetically predicted C4A brain expression for association with depression in the PGC-MDD and UKB samples.

## Results

In total, 207 HLA alleles were imputed in at least 2 PGC-MDD studies, and 102 HLA alleles were imputed in the UKB sample, of which 93 were shared across data sets (Table 1). Variants imputed in either data set were included in the final meta-analysis (minimum effective sample size was 669 for HLA-B-3906 in the PGC-MDD). Four C4 haplotypes (AL-AL, AL-BL, AL-BS, BS) were imputed in all data sets.

**Table 1:** Number of Variants Imputed in ≥2 of the 26 PGG-MDD Studies and the UK Biobank Sample

Gene	PGC-MDD	UKB	Variants in Both PGC-MDD and UKB	Variants in Either PGC-MDD or UKB
*HLA-A*	31	13	13	31
*HLA-B*	48	18	18	48
*HLA-C*	30	14	14	30
*HLA-DPA*	5	3	3	5
*HLA-DPB*	25	11	11	25
*HLA-DQA*	12	7	7	12
*HLA-DQB*	19	12	12	19
*HLA-DRB*	37	24	15	46
Total HLA Alleles	207	102	93	216
C4 Haplotypes	4	4	4	4
SNPs	49,611	47,799	40,561	56,779

C4, complement component 4; HLA, human leukocyte antigen; PGC-MDD, Major Depressive Disorder Working Group of the Psychiatric Genomics Consortium; SNP, single nucleotide polymorphism; UKB, UK Biobank.

There was strong consistency between the frequency and INFO scores of HLA alleles and C4 haplotypes imputed in both the PGC-MDD and UKB samples (correlation *r* = .99 for frequency and *r* = .86 for INFO score) (Figures S2 and S3 in Supplement 1). The INFO score for imputed alleles was higher in the UKB than in the PGC-MDD studies (UKB mean = 0.98, PGC-MDD mean = 0.96), possibly because of the larger HLA reference panel or greater efficiency of the imputation algorithm used.

The genetic correlation between the PGC-MDD and the UKB samples was 0.79 (SE = 0.088). The genome-wide heritability estimate of MDD on the liability scale was 0.09 (SE = 0.01). The estimate of local heritability of MDD across the 5 LD partitions within the MHC was not significant (Figure S4 in Supplement 1, Table S4 in Supplement 1).

**Figure 1:**
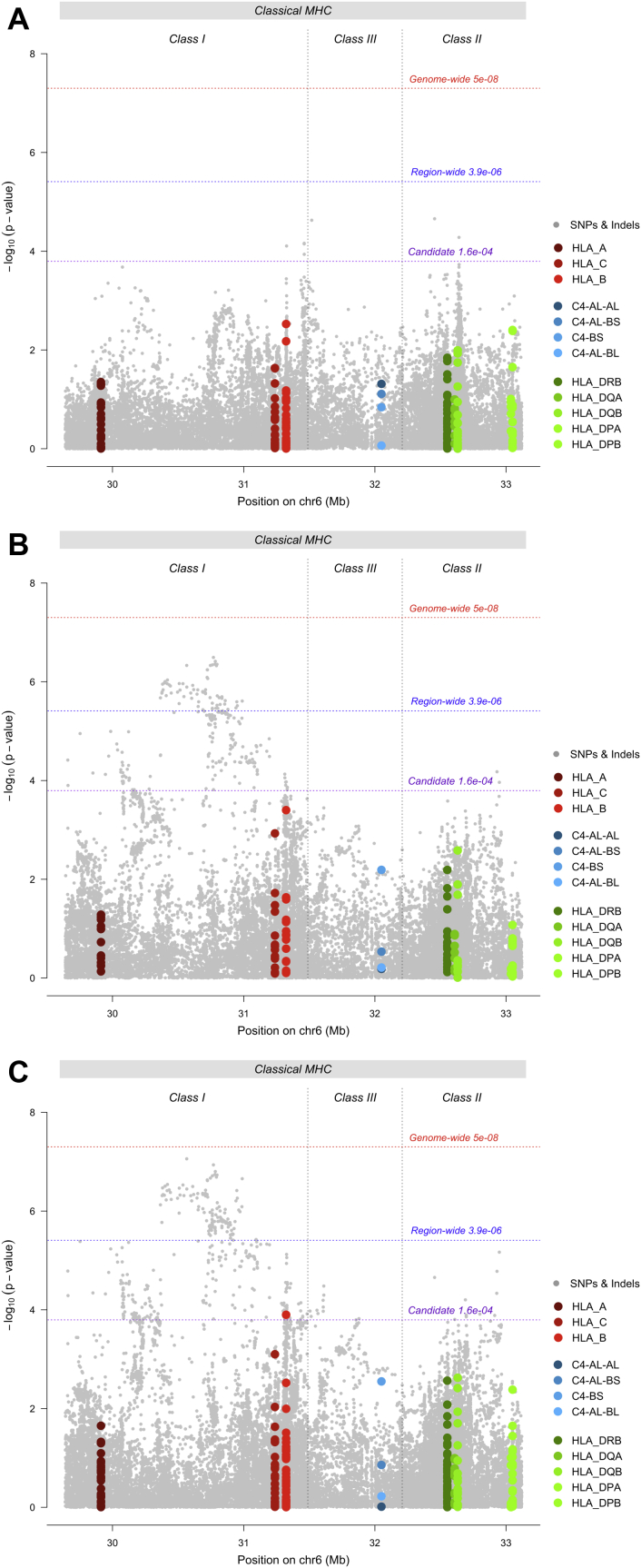
Region-wide Manhattan plots for single nucleotide polymorphisms (SNPs) (gray), human leukocyte antigen (HLA) alleles (HLA-A, HLA-B, and HLA-C [red] and HLA-DPA, HLA-DPB, HLA-DQA, HLA-DQB, and HLA-DRB [green]), and complement component 4 (C4) haplotypes (C4-AL-AL, C4-AL-BS, C4-BS, and C4-AL-BL [blue], where A and B represent the isotype of the *C4* gene, L indicates the long form, and S indicates the short form) in **(A)** Major Depressive Disorder Working Group of the Psychiatric Genomics Consortium (PGC-MDD) studies, **(B)** UK Biobank sample, and **(C)** meta-analysis of PGC-MDD studies and UK Biobank sample. chr, chromosome; Indels, insertions and deletions; MHC, major histocompatibility complex.

Testing for association with depression in the PGC-MDD sample, no HLA allele, C4 haplotype, or SNP surpassed region-wide significance (Figure 1A). In the UKB, no HLA allele or C4 haplotype surpassed region-wide significance (Figure 1B). The allele with strongest evidence for association was HLA-B*08:01 (*p* = 4 × 10^−4^, OR = 0.98, 95% CI = 0.97–0.99). Among SNPs, 70 met region-wide significance (Table S5 in Supplement 2). The variant with the lowest *p* value was a SNP in the classical class I region: rs1264373 (*p* = 3.21 × 10^−7^, OR = 0.97, 95% CI = 0.96–0.98). All variants surpassing region-wide significance were in LD with rs1264373 (0.66 < *r*^*2*^ < 1.00), and rs1264373 was also in LD with the most significant MHC SNP in the PGC-MDD GWAS (17) (rs115507122, *r*^2^ = .63).

In the meta-analysis, no HLA allele or C4 haplotype met region-wide significance; HLA-B*0801 met the candidate threshold (*p* = 1.26 × 10^−4^, OR = 0.98, 95% CI = 0.97–0.99) (Figure 1C). A total of 143 SNPs reached region-wide significance (Table S6 in Supplement 2). The variant with the lowest *p* value was a SNP in the classical class I region: rs9262120 (*p* = 8.74 × 10^−8^, OR = 1.03, 95% CI = 1.02–1.05). This SNP was in LD with the other 142 significant variants (0.44 < *r*^*2*^ < 1.00), and with the most significant SNP within the MHC in the PGC-MDD GWAS (17) (*r*^2^ = 0.66). Low heterogeneity was observed between most variants in the UKB and PGC-MDD meta-analysis; 72% of variants had an *I*^2^ value below 0.25 (Figures S5 and S6 in Supplement 1).

We identified 6 autoimmune diseases with evidence for a bidirectional relationship with depression: Crohn's disease, multiple sclerosis, primary adrenocortical insufficiency, psoriasis vulgaris, systemic lupus erythematosus (SLE), and type 1 diabetes mellitus 9, 13. We identified 14 HLA alleles associated with risk for these autoimmune diseases (*p* < 3.9 × 10^−6^) in European populations 47, 48, 49, 50, 51, 52, 53, 54, 55, 56, 57, 58, with MAF >0.05 in our study. Three HLA alleles had evidence for association with depression after correcting for multiple testing (*p* < .004): HLA-B*08:01 and HLA-DQB1*02:01 (SLE) and HLA-DRB1*03:01 (multiple sclerosis, primary adrenocortical insufficiency, SLE) (Table 2). These alleles were in strong LD with the C4-BS haplotype in the UKB sample; the *r*^2^ values with HLA-B*08:01, HLA-DRB1*03:01, and HLA-DQB1*02:01 were 0.73, 0.70, and 0.68, respectively (Figure S7 in Supplement 1 and Tables S7 and S8 in Supplement 1). Evidence for association with the HLA alleles attenuated after conditioning on C4-BS (*p* = .008, *p* = .2, and *p* = .3, respectively), but the HLA allele showed stronger association than the C4-BS haplotype did (Tables S9–11 in Supplement 1).

**Table 2:** HLA Alleles Associated With Risk for 6 Autoimmune Diseases

Trait (References) [Prevalence in the UKB Sample in Depression Cases, Controls]	HLA Allele	Effect in Autoimmune Disease		PGC-MDD		UKB		Meta-analysis		
		OR	95% CI	Frq	OR	Frq	OR	OR	95% CI	*p* Value
Crohn's Disease 47, 48 [0.46%, 0.39%]	HLA-A*03:01	1.10	1.07–1.15	0.15	0.96	0.14	0.99	0.99	0.98–1.00	.176
	HLA-C*06:02	1.17	1.13–1.23	0.09	1.00	0.09	1.02	1.02	1.00–1.04	.043
	HLA-DRB1*07:01	1.14	1.10–1.18	0.13	1.01	0.14	1.01	1.01	1.00–1.02	.179
	HLA-DRB1*13:02	1.20	1.13–1.28	0.05	0.97	0.04	0.99	0.99	0.97–1.01	.431
Multiple Sclerosis 49, 50 [0.48%, 0.27%]	HLA-DQB1*03:02	1.30	1.23–1.37	0.11	1.00	0.10	1.00	1.00	0.99–1.01	.537
	HLA-DRB1*03:01	1.16	1.10–1.22	0.13	0.95	0.15	0.98	0.98	0.97–0.99	.003^a^
	HLA-DRB1*15:01	3.92	3.74–4.12	0.14	0.98	0.14	1.00	0.99	0.98–1.00	.355
Primary Adrenocortical Insufficiency (Addison's Disease) 51, 52	HLA-DRB1*03:01	2.93	2.12–4.04	0.13	0.95	0.15	0.98	0.98	0.97–0.99	.003^a^
Psoriasis Vulgaris 53, 54 [1.56%, 1.21%]	HLA-A*02:01	1.20	1.08–1.33	0.28	1.01	0.27	1.01	1.01	1.00–1.02	.005
	HLA-C*06:02	3.57	3.12–4.08	0.09	1.00	0.09	1.02	1.02	1.00–1.04	.043
	HLA-DQA1*02:01	1.99	1.74–2.27	0.13	1.01	0.14	1.01	1.01	1.00–1.02	.212
Systemic Lupus Erythematosus 55, 56 [0.21%, 0.11%]	HLA-B*08:01	1.84	1.70–1.99	0.12	0.96	0.14	0.98	0.98	0.97–0.99	1.26 × 10^−4^^a^
	HLA-DQA1*01:02	1.31	1.22–1.40	0.20	0.97	0.19	0.99	0.99	0.98–1.00	.163
	HLA-DQB1*02:01	1.84	1.71–1.99	0.13	0.95	0.15	0.98	0.98	0.97–0.99	.002^a^
	HLA-DRB1*03:01	1.87	1.73–2.02	0.13	0.95	0.15	0.98	0.98	0.97–0.99	.003^a^
Type 1 Diabetes Mellitus 57, 58 [0.48%, 0.35%]	HLA-A*24:02	1.32	NA	0.08	0.97	0.07	1.01	1.00	0.98–1.02	.578
	HLA-DPB1*01:01	1.27	NA	0.05	0.92	0.06	0.99	0.98	0.96–1.00	.067

The prevalence of each autoimmune disease, with the exception of primary adrenocortical insufficiency, which is very rare, within depression cases and controls in the UK Biobank (UKB) sample is shown in the first column. Columns 2–4 show the human leukocyte antigen (HLA) allele association with each autoimmune disease as estimated in the primary studies cited. Remaining columns show the HLA allele association with depression in the Major Depressive Disorder Working Group of the Psychiatric Genomics Consortium (PGC-MDD), UKB, and meta-analysis.

CI, confidence interval; Frq, allele frequency; NA, not available in primary study; OR, odds ratio.

a *p* values met correction for multiple testing.

**Figure 2:**
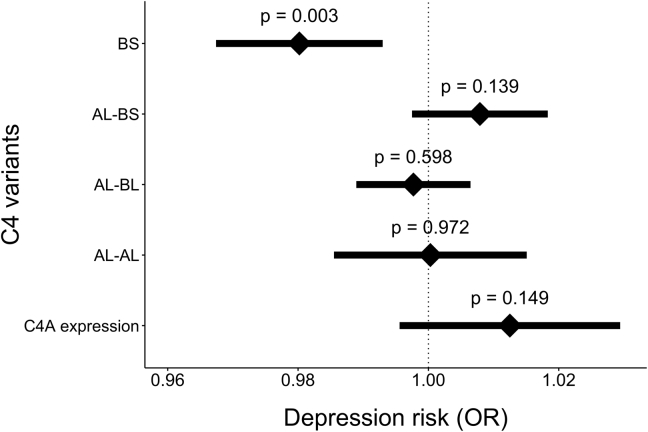
Association of genetically predicted complement component 4A (C4A) brain expression and four C4 haplotypes (C4-BS, C4-AL-BS, C4-AL-BL, and C4AL-AL, where A and B represent the isotype of the *C4* gene, L indicates the long form, and S indicates the short form) in the meta-analysis of Major Depressive Disorder Working Group of the Psychiatric Genomics Consortium (PGC-MDD) studies and UK Biobank sample. Error bars show 95% confidence intervals. OR, odds ratio.

Genetically predicted C4A brain expression was not significantly associated with depression status in the PGC-MDD sample (*p* = .066, OR = 1.06, 95% CI = 1.00–1.13), the UKB sample (*p* = .333, OR = 1.01, 95% CI = 0.99–1.03), or the meta-analysis (*p* = .150, OR = 1.01, 95% CI = 0.99–1.03) (Figure 2 and Table S12 in Supplement 2).

## Discussion

To further understand the mechanisms driving comorbid autoimmunity and depression, we investigated evidence for shared genetic influences in the MHC, a region harboring genetic risk for autoimmune diseases and psychiatric disorders. Our primary aim was to test HLA alleles and C4 haplotypes for association with depression. Under a conservative region-wide significance threshold testing for all variants in the MHC, we found no evidence that HLA alleles, which play a major role in susceptibility to autoimmune diseases, or C4 haplotypes, which are strongly associated with risk for schizophrenia, also confer risk for depression. However, under a candidate threshold, correcting for SNPs within HLA genes, HLA-B*0801 had significant evidence for association with depression status. Conditioning on the C4-BS haplotype resulted in moderate attenuation of signal from HLA-B*0801, indicating independence from C4 haplotypes.

The local heritability estimate of depression in the MHC region was not significant, which is unsurprising given the narrow region considered and the modest SNP heritability for depression across the genome (*h*^2^ = 0.09).

We further explored common HLA alleles associated with autoimmune diseases that have evidence of a bidirectional relationship with depression. The allele with strongest evidence for association with depression was HLA-B*08:01, followed by HLA-DQB1*02:01 and HLA-DRB1*03:01. Previous studies have shown that all 3 HLA alleles increase risk for SLE 55, 56 and that HLA-DRB1*03:01 also increases risk for multiple sclerosis 49, 50 and primary adrenocortical insufficiency 51, 52. In contrast, our findings indicate that HLA-B*08:01, HLA-DQB1*02:01, and HLA-DRB1*03:01 have modest protective effects in depression, indicating that these alleles do not harbor shared risk for autoimmune disease and depression.

Imputation of C4 haplotypes identified 4 common haplotypes, none of which was associated with risk for depression in the PGC-MDD studies, UKB sample, or meta-analysis. These results are in stark contrast to those for schizophrenia, where association with C4 haplotypes accounts for most of the observed SNP association in the HLA region. Our results suggest that C4 does not contribute to the common genetic susceptibility between depression and schizophrenia (genetic correlation *r*_G_ = .34).

At the level of region-wide significance, we detected 70 SNPs associated with depression in the UKB sample and 143 in the meta-analysis. In each case, the top SNP was in moderate to strong LD with other significant variants, indicating a single peak of independent association. We found consistency in SNP signal between our study and the PGC-MDD GWAS of depression (17), with the top SNPs in each study showing moderate to strong LD. This was not unexpected given that our study is a subset of the studies included in the PGC-MDD meta-analysis (17).

The true identity of causal variants within the MHC remains unresolved, and fine-mapping within the MHC is challenging because of the high density of genetic variation and strong LD. Our results strongly suggest that the association signal observed in the MHC in depression 17, 23 does not arise from HLA alleles or C4 haplotypes. These results suggest that any associated variants either are rare or have very modest effect sizes. We note that Howard *et al.* (23) increased power by leveraging a broader phenotyping approach. It is interesting to speculate that the broader depression phenotype captures individuals distressed by physical disease. This interpretation would go some way to explaining signal in the MHC, for which there is evidence for association with more diseases than any other region of the genome (18). However, a more parsimonious explanation could be that MHC signal in depression maps to SNPs or to other genetic loci not imputed in this study. This possibility is highly plausible in light of the fact that the MHC contains more genes than any other region in the human genome (18). Under this scenario, large sample sizes and sequencing may be required to dissect SNP signal within the MHC.

Our findings do not support a role for HLA alleles within the MHC in risk for depression, and cross-trait correlations performed by the PGC-MDD (17) do not support the theory that shared genetic risk for depression and autoimmune diseases is situated outside the MHC. In other efforts to detect genome-wide pleiotropy, Euesden *et al.* (14) found no evidence that polygenic risk scores for rheumatoid arthritis predicted depression status in an independent sample, nor did polygenic risk scores for depression predict autoimmune disease status.

One possibility is that there is a subgroup of individuals enriched for depression and autoimmune risk alleles. Under this scenario, there may be insufficient power to detect the relationship. Identifying, for example, a subgroup of individuals with depression, who are also enriched for autoimmune risk alleles, would go some way to explaining the observed comorbidity between these traits. Furthermore, identifying a subtype of depression, e.g., an “immune-related” depression group, would help to dissect heterogeneity in the depression phenotype.

In summary, this study is the first to interrogate the involvement of HLA alleles and C4 haplotypes in depression risk, and we find no evidence that either type of genetic variant plays a major role in susceptibility for depression. In contrast, the 3 HLA alleles that showed nominal significance in our study conferred modest protective effects for depression. Furthermore, the strong association with C4 alleles that is seen in cases of schizophrenia is absent in cases of depression. Large sample sizes and regional sequence data may be required to dissect SNP signal within the MHC.

## Supplementary Material

Supplement 2

Key Resources Table
