# Supplementary material for: Classical Human Leukocyte Antigen Alleles and C4 Haplotypes Are Not Significantly Associated With Depression
Source: Biol Psychiatry. 2020 Mar 1;87(5):419–30. doi: 10.1016/j.biopsych.2019.06.031 (PMC7001040; doi:10.1016/j.biopsych.2019.06.031)
Supplement: Supplement 1 [file mmc1.pdf]

# Classical Human Leukocyte Antigen Alleles and Complement Component 4 Haplotypes Are Not Significantly Associated With Depression

## *Supplement 1*

## Table of Contents

**Supplementary note on differences between QC and analysis pipelines in the PGC and UKB.........2**
**Quality control.........2**
**HLA imputation.........2**
**Statistical analyses.........2**
**Supplementary Figure S1:** Binomial distribution of residuals ......... 3
**Supplementary Figure S2: correlation between frequencies in the PGC and UKB.........4**
**Supplementary Figure S3: correlation between info scores in the PGC and UKB .........5**
**Supplementary Figure S4: SNP heritability of MDD partitioned across the genome .........6**
**Supplementary Table S4: SNP heritability of MDD partitioned across the MHC .........6**
**Supplementary Figure S5: effect size heterogeneity for variants in the meta-analysis .........7**
**Supplementary Figure S6: effect size heterogeneity for HLA alleles in the meta-analysis.........8**
**Supplementary Figure S7: LD between common C4 haplotypes and HLA alleles.........9**
**Supplementary Tables S7 and S8: LD between common C4 haplotypes and HLA alleles.........10**
**Supplementary Tables S9, S10 and S11: Conditional analyses .........11**
**Supplementary References .........13**

See Supplement 2 (Excel file) for Supplementary Tables S1, S2, S3, S5, S6, and S12.

# Supplementary note on differences between QC and analysis pipelines in the PGC and UKB

## Quality control

Our study leverages data-sets that have previously undergone quality control by the PGC Statistical Analysis Group using the ricopili pipeline[1] and the Statistical Genetics Unit of King's College London for UKB data. The QC pipelines between the PGC and UKB diverge on the following thresholds: SNP missingness before individual QC (PGC < 0.05, UKB < 0.02), MAF (PGC > 0.05, UKB > 0.01), HWE (PGC > $10^{-10}$ (cases) and > $10^{-6}$ (controls), UKB > $10^{-8}$), relatedness (PGC > 0.2, UKB > 0.044 [3rd degree relatedness in the UKB[2]]). We did not see the need to re-compute quality control to adjust for minor differences between samples, which are often observed in meta-analytic studies.

## HLA imputation

Different software packages were used to impute HLA alleles in the UKB and PGC samples. Imputation was performed by the UKB core analytical team using a commercial software package; HLA*IMP:02 software[2]. We imputed the HLA alleles in the PGC using an open source software; SNP2HLA software[3]. We observed strong correlation between the frequency and info score of imputed variants in the UKB and PGC (r=0.99 and r=0.86 respectively, Supplementary Figures S2 and S3), and therefore decided there would be little benefit to repeating HLA imputation in the UKB using the SNP2HLA software.

## Statistical analyses

In the PGC we used logistic regression to test the association of SNPs, HLA alleles and C4 haplotypes with depression case/control status, controlling for 6 principal components. In the UKB, we first regressed 6 principal components, batch and centre on the depression phenotype using logistic regression. We checked for a binomial distribution of residuals (Supplementary Figure S1) and performed linear regression on the residuals to test for association of SNPs, HLA alleles and C4 haplotypes with depression. We took this approach due to a computational limitation of PLINK (version 1.9 and version 2.0)[4] when analyzing this large number of covariates in a logistic regression model (n=128). Using linear regression is computationally efficient and retains consistency with other UK Biobank analyses from our research group[5], although analysis using logistic regression in other software packages such as R would have been possible.

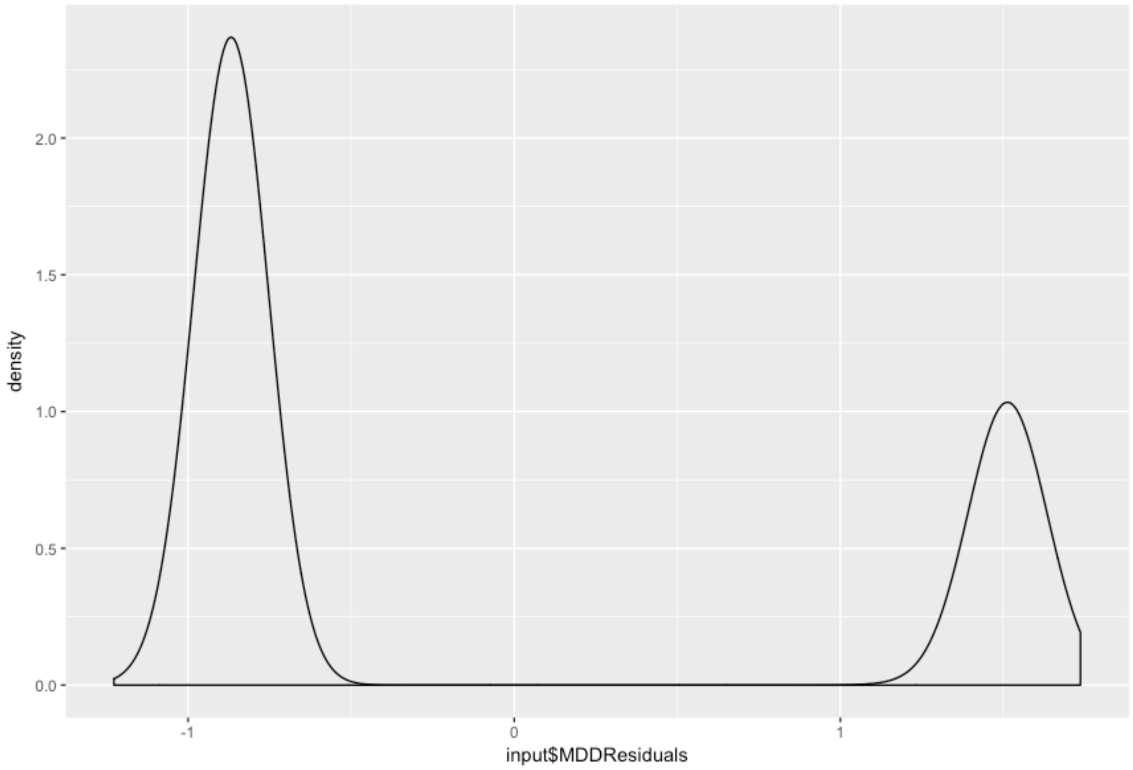


**Supplementary Figure S1:** Binomial distribution of residuals from logistic regression of 6 principal components, batch and centre on the depression phenotype in the UKB.

## Supplementary Figure S2: correlation between frequencies in the PGC and UKB

In Supplementary Figure S2 frequencies are expressed as -$\log_{10}$ to highlight rare alleles.

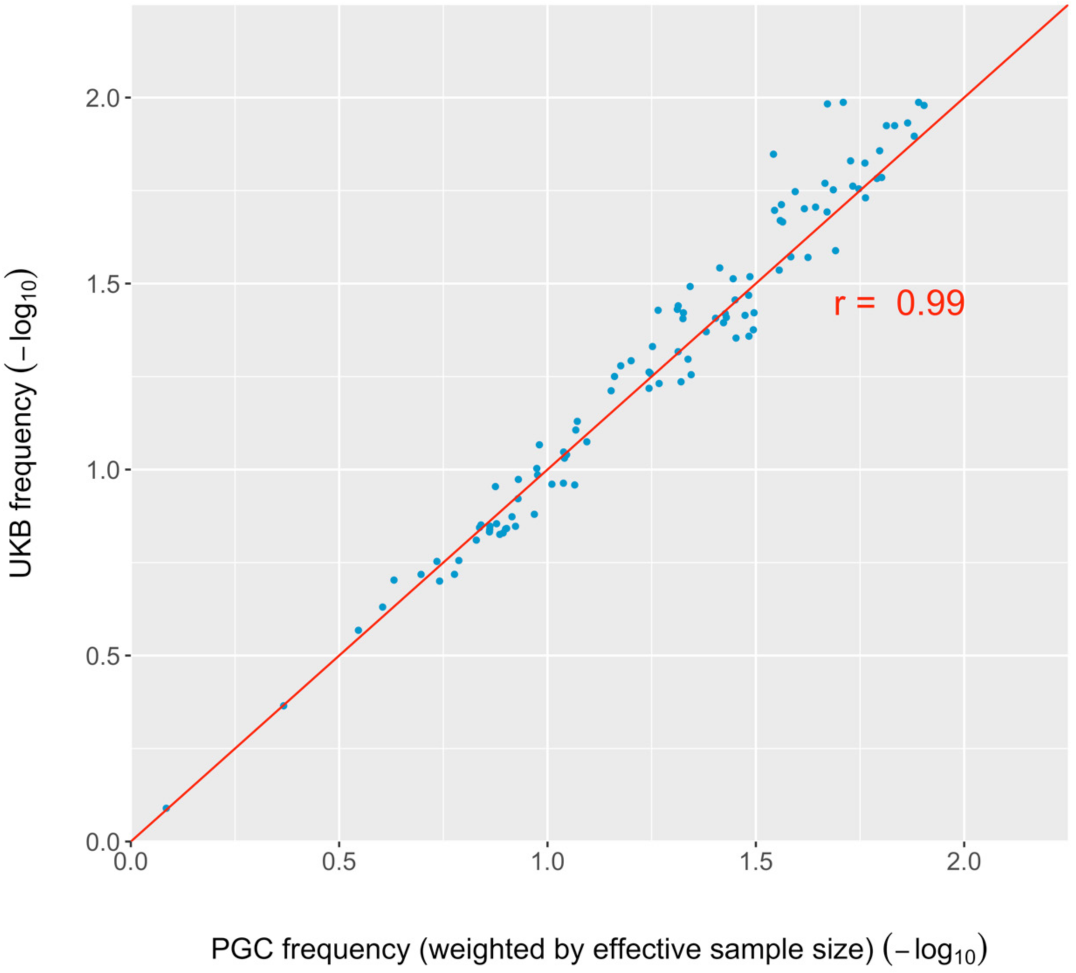


**Supplementary Figure S2:** Correlation between frequency (-$\log_{10}$) of HLA alleles and C4 haplotypes common to the PGC and UKB, weighted by effective sample size in the PGC.

## Supplementary Figure S3: correlation between info scores in the PGC and UKB

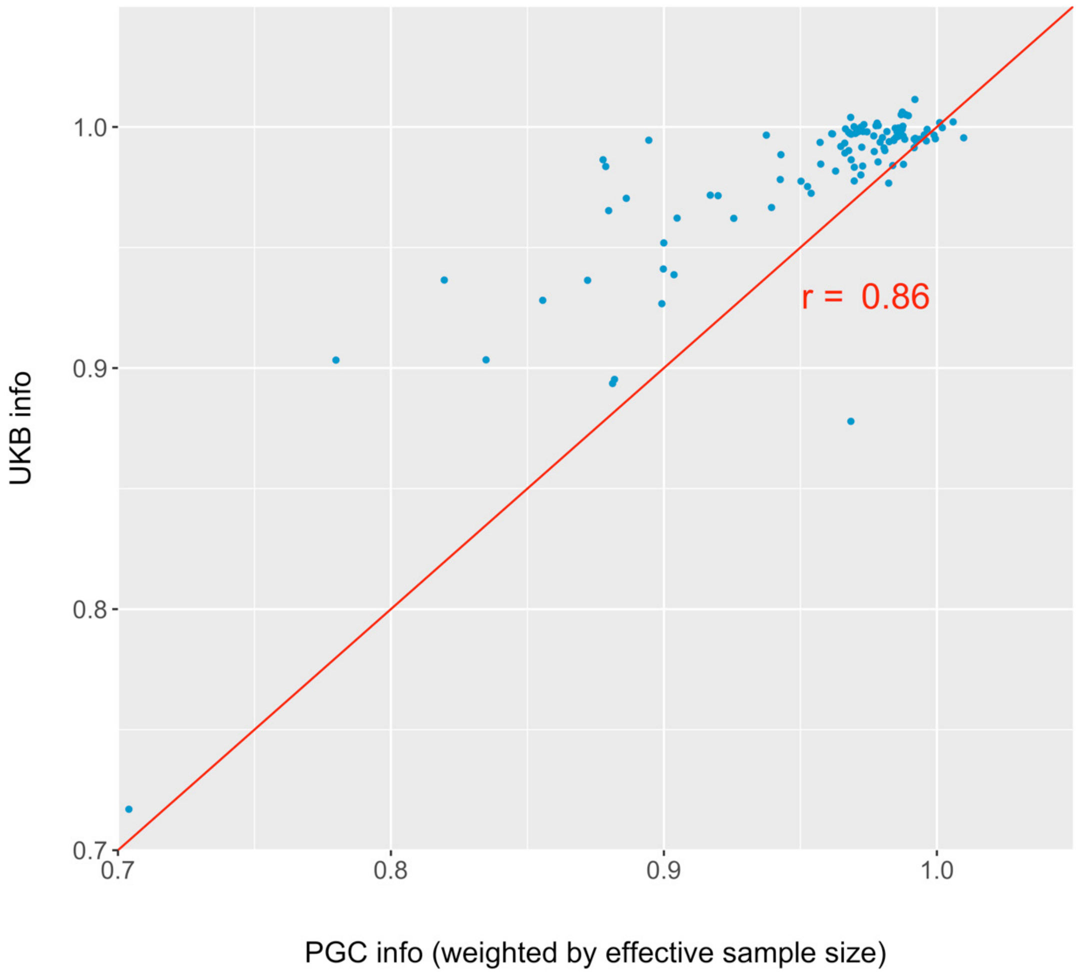


**Supplementary Figure S3:** Correlation between info scores for HLA alleles and C4 haplotypes common to the PGC and UKB, weighted by effective sample size in the PGC.

## Supplementary Figure S4: SNP heritability of MDD partitioned across the genome

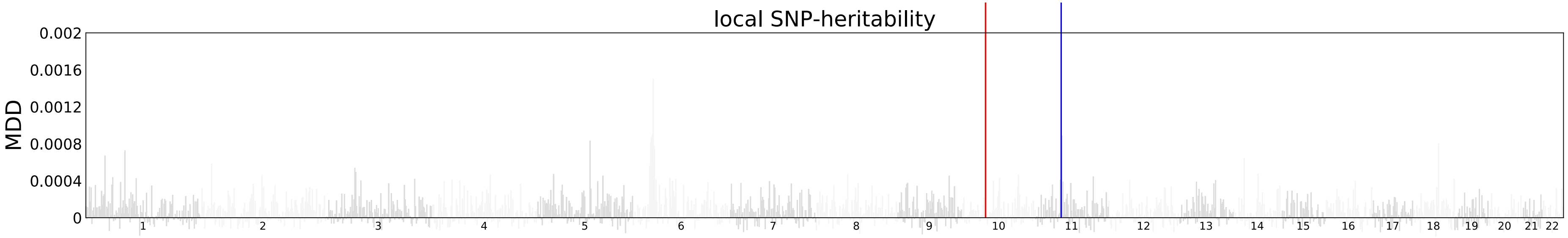


**Supplementary Figure S4:** SNP heritability estimates for MDD using summary statistics from the PGC-MDD GWAS (excluding 23andMe data). Heritability is shown on the y-axis and chromosomes/loci are shown on the x-axis. Coloured vertical lines denote loci with significant SNP heritability.

## Supplementary Table S4: SNP heritability of MDD partitioned across the MHC

| Chromosome | Start | End | N SNPs | k | Local $h^2$ | Variance | SE | z-score | p-value |
|---|---|---|---|---|---|---|---|---|---|
| 6 | 28917608 | 29737971 | 199 | 17 | 0.00090 | 1.26E-07 | 0.00036 | 2.5226 | 0.00582 |
| 6 | 29737971 | 30798168 | 592 | 20 | 0.00150 | 7.06E-07 | 0.00084 | 1.7880 | 0.03689 |
| 6 | 30798168 | 31571218 | 1588 | 20 | 0.00077 | 5.49E-07 | 0.00074 | 1.0432 | 0.14843 |
| 6 | 31571218 | 32682664 | 5404 | 20 | 0.00017 | 4.23E-07 | 0.00065 | 0.2595 | 0.39762 |
| 6 | 32682664 | 33236497 | 734 | 20 | 0.00041 | 4.86E-07 | 0.00070 | 0.5948 | 0.27598 |

**Supplementary Table S4:** Local SNP heritability estimates for MDD within five LD partitions across the MHC. From left to right columns denote: chromosome, start position of the partition, end position of the partition, number of SNPs in the partition, k = number of eigenvectors used in truncated singular value decomposition, local SNP heritability estimate, variance of the local SNP heritability, standard error, z-score, p-value.

## Supplementary Figure S5: effect size heterogeneity for variants in the meta-analysis

72% of variants had an $I^2$ value below 25% in the meta-analysis.

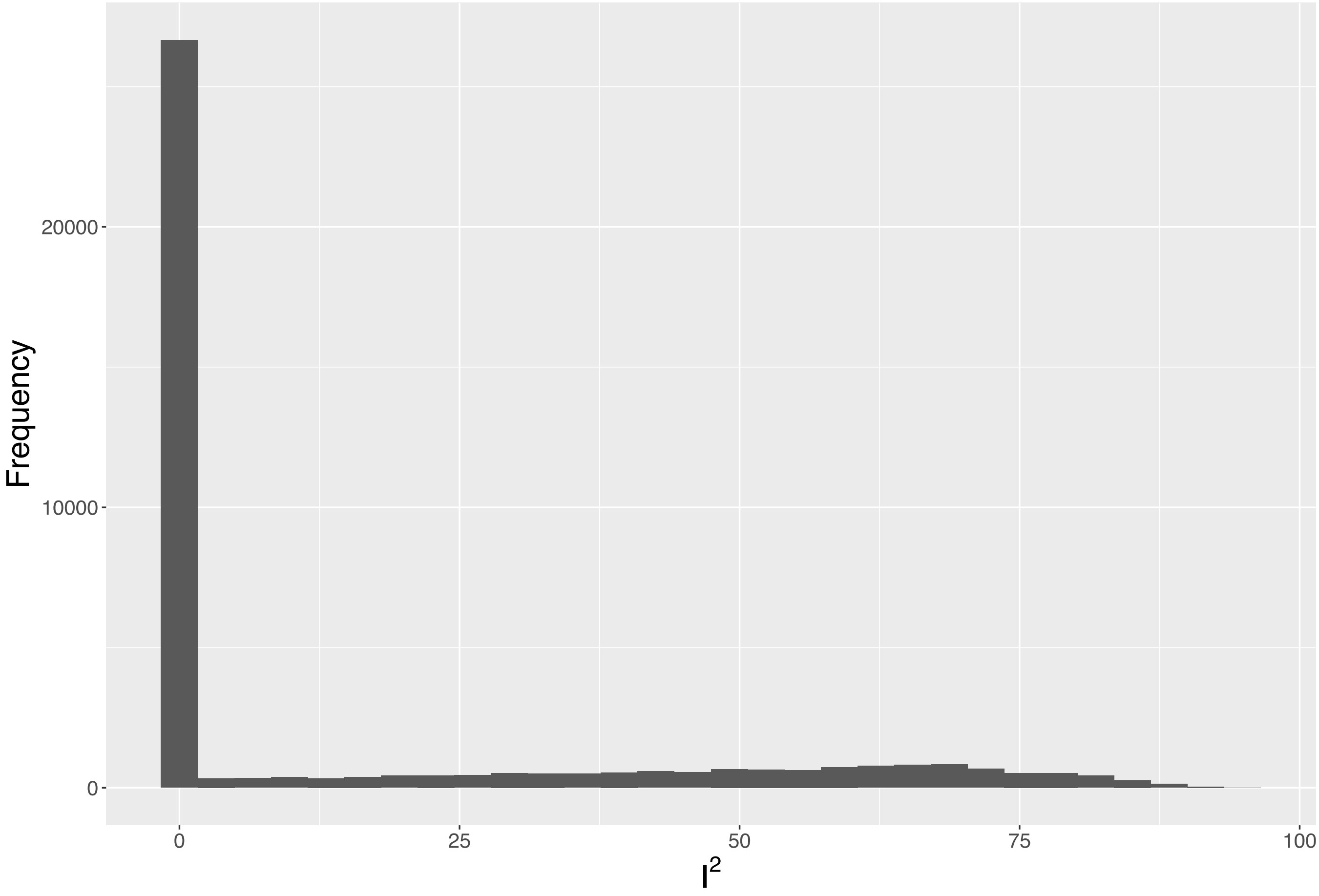


**Supplementary Figure S5:** Frequency of $I^2$ values across all variants in the meta-analysis.

## Supplementary Figure S6: effect size heterogeneity for HLA alleles in the meta-analysis

71% of HLA alleles had an $I^2$ value below 25% in the meta-analysis.

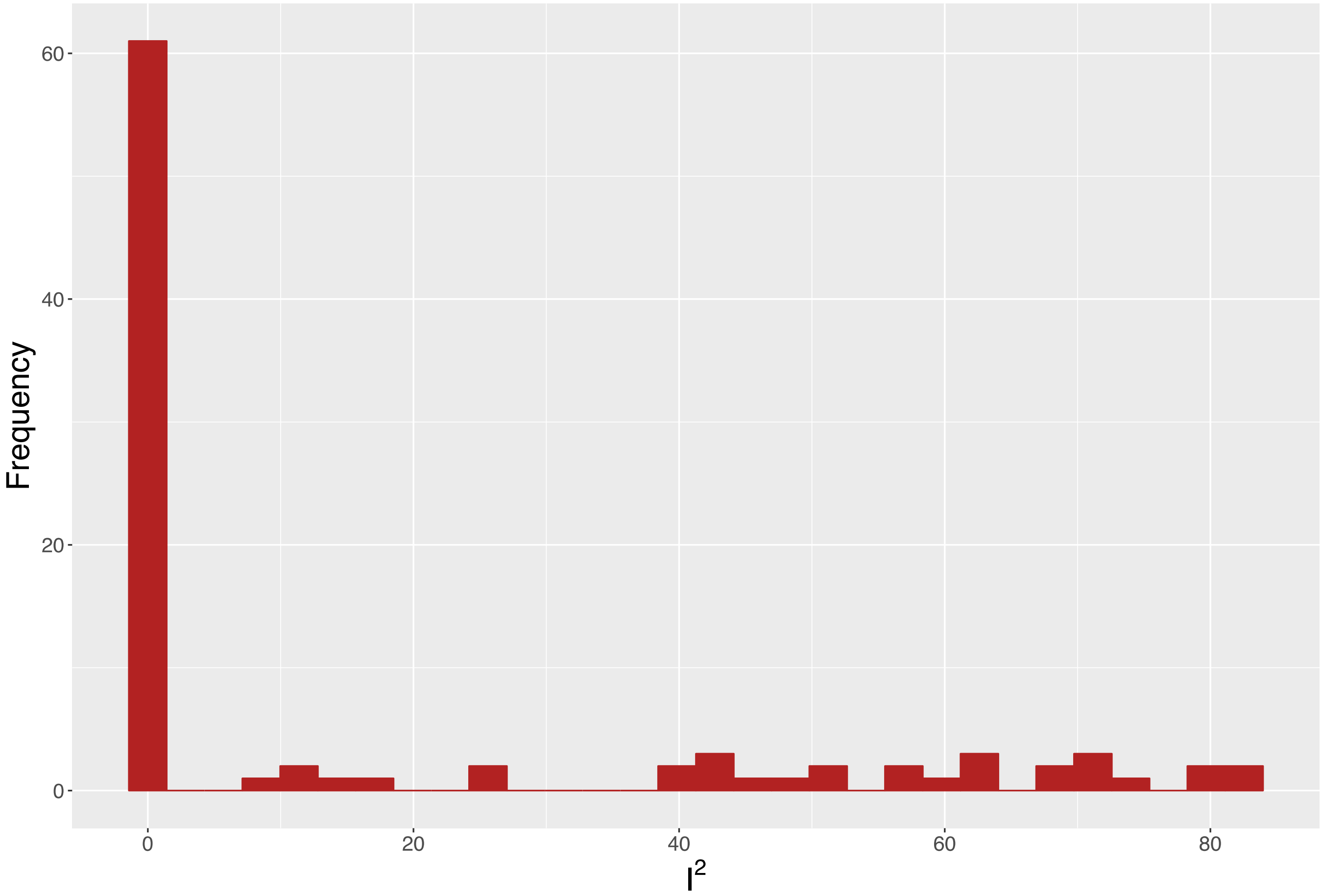


**Supplementary Figure S6:** Frequency of $I^2$ values across HLA alleles in the meta-analysis.

## Supplementary Figure S7: LD between common C4 haplotypes and HLA alleles

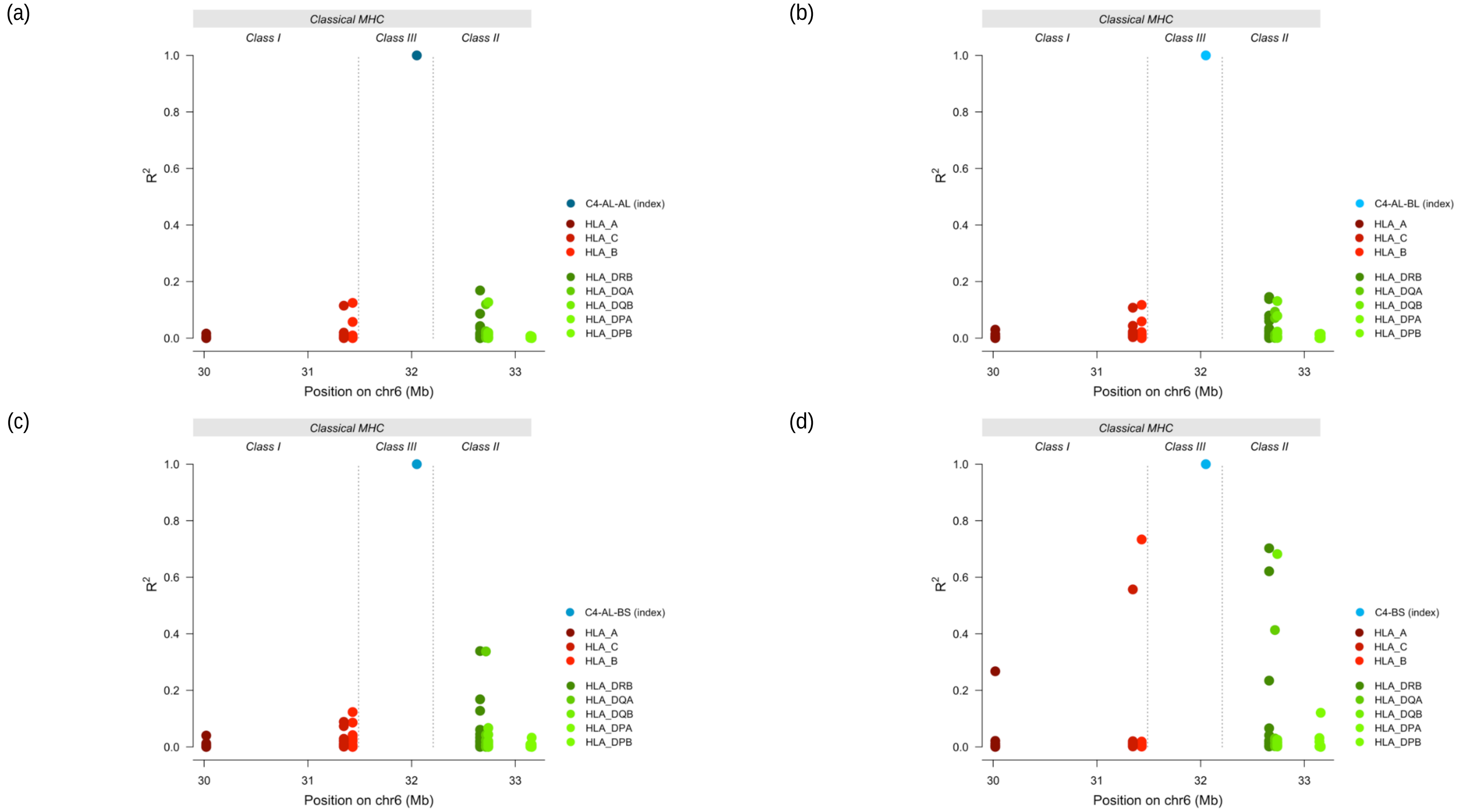


**Supplementary Figure S7:** Region-wide plots for linkage-disequilibrium (LD) between C4 haplotypes (blue) and HLA alleles (red/green) in the UK Biobank. One of each of the common C4 haplotypes is used as the index variant in each plot: (a) C4-AL-AL, (b) C4-AL-BL, (c) C4-AL-BS, and (d) C4-BS.

## Supplementary Tables S7 and S8: LD between common C4 haplotypes and HLA alleles

Supplementary tables S7 and S8 show HLA alleles in LD with C4 haplotypes ($R^2 > 0.2$) in the UKB (grey columns) and evidence for association between those HLA alleles and depression in the PGC, UKB and meta-analysis (blue columns).

| LD information | | | | | | | | | | HLA association with depression | | | | | |
|---|---|---|---|---|---|---|---|---|---|---|---|---|---|---|---|
| Index C4 haplotype | | | | HLA LD partner | | | | | | PGC | | UKB | | Meta-analysis | |
| CHR | BP | HAPLOTYPE | MAF | CHR | BP | HLA ALLELE | MAF | $R^2$ | D'prime | OR | P | OR | P | OR | P |
| 6 | 32050001 | C4-AL-BS | 0.24 | 6 | 32660042 | HLA_DRB1_0701 | 0.14 | 0.34 | 0.79 | 1.01 | 0.772 | 1.01 | 0.189 | 1.01 | 0.179 |
| 6 | 32050001 | C4-AL-BS | 0.24 | 6 | 32716284 | HLA_DQA1_0201 | 0.14 | 0.34 | 0.78 | 1.01 | 0.792 | 1.01 | 0.223 | 1.01 | 0.212 |

**Supplementary Table S7:** HLA alleles with an $R^2$ value > 0.2 with C4-AL-BS in the UKB.

| LD information | | | | | | | | | | HLA association with depression | | | | | |
|---|---|---|---|---|---|---|---|---|---|---|---|---|---|---|---|
| Index C4 haplotype | | | | HLA LD partner | | | | | | PGC | | UKB | | Meta-analysis | |
| CHR | BP | HAPLOTYPE | MAF | CHR | BP | HLA ALLELE | MAF | $R^2$ | D'prime | OR | P | OR | P | OR | P |
| 6 | 32050000 | C4-BS | 0.13 | 6 | 31431272 | **HLA_B_0801** | 0.14 | 0.73 | 0.89 | 0.96 | 0.097 | 0.98 | 3.98e-04 | 0.98 | 1.26e-04 |
| 6 | 32050000 | C4-BS | 0.13 | 6 | 32660042 | **HLA_DRB1_0301** | 0.15 | 0.70 | 0.90 | 0.95 | 0.017 | 0.98 | 0.015 | 0.98 | 0.003 |
| 6 | 32050000 | C4-BS | 0.13 | 6 | 32739039 | **HLA_DQB1_0201** | 0.15 | 0.68 | 0.89 | 0.95 | 0.018 | 0.98 | 0.013 | 0.98 | 0.002 |
| 6 | 32050000 | C4-BS | 0.13 | 6 | 32660042 | HLA_DRB3_0101 | 0.17 | 0.62 | 0.90 | NA | NA | 0.99 | 0.022 | 0.99 | 0.022 |
| 6 | 32050000 | C4-BS | 0.13 | 6 | 31346171 | HLA_C_0701 | 0.18 | 0.56 | 0.89 | 0.98 | 0.393 | 0.98 | 0.001 | 0.98 | 0.001 |
| 6 | 32050000 | C4-BS | 0.13 | 6 | 32716284 | HLA_DQA1_0501 | 0.23 | 0.41 | 0.90 | 0.99 | 0.541 | 0.99 | 0.138 | 0.99 | 0.108 |
| 6 | 32050000 | C4-BS | 0.13 | 6 | 30019970 | HLA_A_0101 | 0.19 | 0.27 | 0.65 | 1.00 | 0.991 | 0.99 | 0.065 | 0.99 | 0.081 |
| 6 | 32050000 | C4-BS | 0.13 | 6 | 32660042 | HLA_DRB3_9901 | 0.35 | 0.23 | 0.91 | NA | NA | 1.01 | 0.238 | 1.01 | 0.238 |

**Supplementary Table S8:** HLA alleles with an $R^2$ value > 0.2 with C4-BS in the UKB. Highlighted in red are the three HLA alleles that were significantly associated with depression in the follow-up analyses of HLA alleles that confer risk for autoimmune diseases that have a bi-directional relationship with depression. NA = allele not imputed in the PGC.

## Supplementary Tables S9, S10 and S11: Conditional analyses

Tables S9, S10 and S11 show the results of conditional analyses in the PGC, UKB and meta-analysis, respectively. The first four rows in each table show evidence for association of HLA_B_0801, HLA_DQB1_0201, HLA_DRB1_0301 and C4-BS with depression. The last three rows in each table show estimates for the same HLA alleles when C4-BS is included as a covariate in the model.

| **Model** | **HLA_B_0801** | | | **HLA_DQB1_0201** | | | **HLA_DRB1_0301** | | | **C4-BS** | | |
|---|---|---|---|---|---|---|---|---|---|---|---|---|
| | **OR** | **SE** | **P-value** | **OR** | **SE** | **P-value** | **OR** | **SE** | **P-value** | **OR** | **SE** | **P-value** |
| **HLA_B_0801** | 0.96 | 0.024 | 0.097 | | | | | | | | | |
| **HLA_DQB1_0201** | | | | 0.95 | 0.024 | 0.018 | | | | | | |
| **HLA_DRB1_0301** | | | | | | | 0.95 | 0.024 | 0.017 | | | |
| **C4-BS** | | | | | | | | | | 0.96 | 0.027 | 0.144 |
| **HLA_B_0801 + C4-BS** | 0.96 | 0.042 | 0.313 | | | | | | | 0.98 | 0.052 | 0.629 |
| **HLA_DQB1_0201 + C4-BS** | | | | 0.92 | 0.039 | 0.040 | | | | 1.03 | 0.049 | 0.525 |
| **HLA_DRB1_0301 + C4-BS** | | | | | | | 0.92 | 0.040 | 0.049 | 1.04 | 0.050 | 0.469 |

**Supplementary Table S9:** Conditional analyses in the PGC cohort.

| **Model** | **HLA_B_0801** | | | **HLA_DQB1_0201** | | | **HLA_DRB1_0301** | | | **C4-BS** | | |
|---|---|---|---|---|---|---|---|---|---|---|---|---|
| | **OR** | **SE** | **P-value** | **OR** | **SE** | **P-value** | **OR** | **SE** | **P-value** | **OR** | **SE** | **P-value** |
| **HLA_B_0801** | 0.98 | 0.007 | 3.98e-04 | | | | | | | | | |
| **HLA_DQB1_0201** | | | | 0.98 | 0.007 | 0.013 | | | | | | |
| **HLA_DRB1_0301** | | | | | | | 0.98 | 0.007 | 0.015 | | | |
| **C4-BS** | | | | | | | | | | 0.98 | 0.007 | 0.006 |
| **HLA_B_0801 + C4-BS** | 0.97 | 0.013 | 0.014 | | | | | | | 1.01 | 0.013 | 0.482 |
| **HLA_DQB1_0201 + C4-BS** | | | | 0.99 | 0.012 | 0.506 | | | | 0.99 | 0.012 | 0.346 |
| **HLA_DRB1_0301 + C4-BS** | | | | | | | 1.00 | 0.012 | 0.687 | 0.99 | 0.013 | 0.253 |

**Supplementary Table S10:** Conditional analyses in the UKB cohort.

| Model | HLA_B_0801 | | | HLA_DQB1_0201 | | | HLA_DRB1_0301 | | | C4-BS | | |
|---|---|---|---|---|---|---|---|---|---|---|---|---|
| | OR | SE | P-value | OR | SE | P-value | OR | SE | P-value | OR | SE | P-value |
| **HLA_B_0801** | 0.98 | 0.007 | 1.260e-04 | | | | | | | | | |
| **HLA_DQB1_0201** | | | | 0.98 | 0.006 | 0.002 | | | | | | |
| **HLA_DRB1_0301** | | | | | | | 0.98 | 0.006 | 0.003 | | | |
| **C4-BS** | | | | | | | | | | 0.98 | 0.007 | 0.003 |
| **HLA_B_0801 + C4-BS** | 0.97 | 0.012 | 0.008 | | | | | | | 1.01 | 0.013 | 0.576 |
| **HLA_DQB1_0201 + C4-BS** | | | | 0.99 | 0.011 | 0.220 | | | | 0.99 | 0.012 | 0.448 |
| **HLA_DRB1_0301 + C4-BS** | | | | | | | 0.99 | 0.012 | 0.340 | 0.99 | 0.012 | 0.352 |

**Supplementary Table S11**: Conditional analyses in the PGC and UKB meta-analysis.

## Supplementary References

1. Wray, N. R. *et al.* Genome-wide association analyses identify 44 risk variants and refine the genetic architecture of major depression. *Nature Genetics* 50, 668–681 (2018).
2. Bycroft, C. *et al.* Genome-wide genetic data on ~500,000 UK Biobank participants. *bioRxiv* 166298 (2017). doi:10.1101/166298
3. Jia, X. M. *et al.* Imputing Amino Acid Polymorphisms in Human Leukocyte Antigens. *PLOS ONE* 8, 1–10 (2013).
4. Chang, C. C. *et al.* Second-generation PLINK: rising to the challenge of larger and richer datasets. *GigaScience* 4, 1–16 (2015).
5. Coleman, J. R. I. *et al.* Genome-wide gene-environment analyses of depression and reported lifetime traumatic experiences in UK Biobank. *bioRxiv* 247353 (2018). doi:10.1101/247353
